# Antibiotic treatment after appendectomy for acute complicated appendicitis to prevent intrabdominal abscess and wound infections

**DOI:** 10.1007/s00423-024-03367-z

**Published:** 2024-06-08

**Authors:** Bruno Leonardo Bancke Laverde, Matthias Maak, Melanie Langheinrich, Stephan Kersting, Axel Denz, Christian Krautz, Georg F. Weber, Robert Grützmann, Maximilian Brunner

**Affiliations:** 1 Department of General and Visceral Surgery, Friedrich-Alexander-University Erlangen-Nürnberg (FAU), Krankenhausstraße 12, 91054 Erlangen, Germany; 2 Department of General, Visceral, Thoracic and Vascular Surgery, University Greifswald, Ferdinand- Sauerbruch-Straße, 17475 Greifswald, Germany

**Keywords:** Complicated appendicitis, Appendectomy, Postoperative antibiotic therapy, Intraabdominal abscess, Wound infection

## Abstract

**Introduction:**

The purpose of this analysis was to investigate the most appropriate duration of postoperative antibiotic treatment to minimize the incidence of intraabdominal abscesses and wound infections in patients with complicated appendicitis.

**Materials and methods:**

In this retrospective study, which included 396 adult patients who underwent appendectomy for complicated appendicitis between January 2010 and December 2020 at the University Hospital Erlangen, patients were classified into two groups based on the duration of their postoperative antibiotic intake: ≤ 3 postoperative days (group 1) vs. ≥ 4 postoperative days (group 2). The incidence of postoperative intraabdominal abscesses and wound infections were compared between the groups. Additionally, multivariate risk factor analysis for the occurrence of intraabdominal abscesses and wound infections was performed.

**Results:**

The two groups contained 226 and 170 patients, respectively. The incidence of postoperative intraabdominal abscesses (2% vs. 3%, *p* = 0.507) and wound infections (3% vs. 6%, *p* = 0.080) did not differ significantly between the groups. Multivariate analysis revealed that an additional cecum resection (OR 5.5 (95% CI 1.4–21.5), *p* = 0.014) was an independent risk factor for intraabdominal abscesses. A higher BMI (OR 5.9 (95% CI 1.2–29.2), *p* = 0.030) and conversion to an open procedure (OR 5.2 (95% CI 1.4–20.0), *p* = 0.016) were identified as independent risk factors for wound infections.

**Conclusion:**

The duration of postoperative antibiotic therapy does not appear to influence the incidence of postoperative intraabdominal abscesses and wound infections. Therefore, short-term postoperative antibiotic treatment should be preferred.

## Introduction

Appendicitis is one of the most frequent reasons for emergency room visits, with an incidence of 124 cases per 100,000 people in Germany. The lifetime risk of developing appendicitis is approximately 7–8% [1, 2]. Acute appendicitis can be categorized into two forms: uncomplicated and complicated. Complicated appendicitis is seen in approximately 30% of all patients that undergo appendectomy and is characterized by transmural inflammation with features like necrosis (gangrenous), perforation, and/or the formation of abscesses in the pelvic or intraabdominal region [3]. Patients with complicated appendicitis tend to experience more postoperative morbidity including infectious complications, longer hospital stays and a higher likelihood of readmission [4–10].

As an inflammatory condition, antibiotics play a relevant role in appendicitis treatment. There is an ongoing debate about whether acute appendicitis can be managed solely with antibiotic therapy. The literature presents promising data for antibiotic treatment of uncomplicated appendicitis, but with a notable risk of recurrence [11–15]. Therefore, emergency appendectomy remains the standard approach, particularly in cases of complicated appendicitis [16]. Furthermore, antibiotic therapy serves as an additional postoperative treatment following appendectomy, with the primary aim of preventing postoperative infectious complications [17, 18]. The most common postoperative infectious complications include the development of intraabdominal abscesses (occurring in about 1.5 − 7.9% of patients after appendectomy) and postoperative wound infections (ranging from 3.4 to 10.0%) [17–19]. However, the available evidence on postoperative antibiotic therapy is limited, with substantial debates regarding the optimal duration of antibiotic treatment [20]. Benefits of implementing antibiotic restriction encompass decreased hospital stays and minimized antibiotic-related adverse effects including diarrhoea, nausea, allergies, thrombophlebitis and Clostridium difficile infection. Moreover, healthcare costs could be reduced by restricted antibiotic use [21, 22].

The objective of this study is to compare outcomes among patients with complicated appendicitis who received different durations of postoperative antibiotic therapy after appendectomy, with a particular focus on assessing infectious complications.

## Material and method

This retrospective study included 1,570 patients aged 18 or older who underwent appendectomy +/- coecal resection for acute appendicitis at the Department of General and Visceral Surgery of the University Hospital Erlangen between January 2010 and December 2020. Patients who required ileocoecal resection for local source control were not considered for this analysis due to their different risk profile stemming from the need for an anastomosis. Based on intraoperative findings, acute appendicitis was classified as either uncomplicated or complicated. Complicated appendicitis was defined as gangrenous or/and perforated appendicitis with or without peritonitis and/or perityphlitic abscess. Consequently, 1,074 patients with uncomplicated appendicitis were excluded from the analysis, resulting in a study cohort of 394 patients with complicated appendicitis.

The study cohort was divided into two groups based on the duration of their postoperative antibiotic therapy: Group 1 included patients who received at least a single intraoperative dose of antibiotics and an additional postoperative antibiotic therapy for a maximum of three days, while group 2 had additionally postoperative antibiotic therapy for four or more days. Stratification was based on continuous postoperative antibiotic therapy initiated immediately after surgery. Patients who required antibiotics again due to complications after discontinuing postoperative antibiotics were categorized based on their initial duration of postoperative antibiotic therapy. The cut-off of three days of antibiotic therapy was chosen according to comparable studies in the literature [17].

Primary endpoint of the study was the occurrence of an intraabdominal abscess and of a postoperative wound infection in the two groups as well as their risk factors.

Data regarding patient demographics, preoperative diagnostics, intraoperative and histopathological findings, as well as the postoperative course were extracted from the clinical information system for group comparisons and subsequent statistical analysis. Based on the intraoperative findings, the patients were classified according to the AAST classification [23]. Morbidity was defined as any deviation from the ordinary postoperative course and classified according to the Clavien-Dindo Classification [24]. An intraabdominal abscess was defined as any intra-abdominal fluid collection that required treatment with antibiotic therapy, interventional drainage or re-surgery. Wound infection was defined was defined according to wound healing CDC definition for superficial incisional surgical site infections [25]. The follow-up time of the patients included 90 days after surgery.

This study was approved by the FAU University Ethical Commission (22-157-Br).

### Antibiotic and surgical standards

All patients received a single intravenous dose of either Ceftriaxone (2 g) or Cefotaxime (2 g) and Metronidazole (500 mg) as antibiotic prophylaxis. The therapeutic antibiotic regimen was continued as per the operative prescription provided by the operating surgeon. The standard postoperative antibiotic regimen consisted of either Cefotaxime (2 g, 3 times a day) and Metronidazole (500 mg, 3 times a day) or piperacillin/tazobactam (4.5 g, 3 times a day). Both antibiotic regimens were administered intravenously as standard during the hospital stay. Antibiotic therapy was continued orally after discharge in 61 patients (15%). The duration of antibiotic therapy was determined collaboratively by the surgeon and the attending physicians on the ward, considering the results of the intraoperative swab and the patient’s clinical condition.

The standard procedure for appendectomy involved a laparoscopic 3-port appendectomy. However, the choice of surgical approach was made by the operating surgeon. During initial study period, an open approach was more commonly chosen. The appendix removal method (Roeder loop or stapler) was selected based on the surgeon’s discretion. In cases of severe inflammation at the appendix base, a coecal pole resection was performed. All surgeries were conducted either by a specialist surgeon or a resident. Residents were always supervised by a specialist surgeon experienced in both open and laparoscopic procedures.

### Statistical analysis

We conducted the statistical analyses using SPSS Statistics (Version 28.0, IBM). Ordinal and metric data were compared using the Mann-Whitney U test or Student’s t-test, while the chi-square test was employed for categorical data. Statistical significance was defined as *p* < 0.05. All assessed baseline parameters as well as the use of postoperative antibiotics were initially tested univariately for their association with wound infection and intraabdominal abscess. For better clarity, only the *p*-values were provided for the univariate analysis. Any parameters found to have a significant association in univariate analysis, along with the duration of antibiotic therapy, were subsequently included in the multivariate analysis. For the multivariately tested values, the odds ratio, the confidence interval, and the *p*-value were provided.

## Results

### Baseline patient characteristics

During the study period, a total of 396 patients (median age: 51 years, 44% female) met the inclusion criteria. Out of these, 226 patients were categorized into group 1 and 170 patients into group 2.

Regarding the baseline patient characteristics, the groups exhibited significant differences in the following parameters: Patients with a duration of postoperative antibiotic therapy ≥ 4 days were older (53 vs. 48 years, *p* = 0.049), had a worse ASA score (*p* = 0.012), a higher preoperative CRP value (160 vs. 90 mg/l, *p* < 0.001), a lower hemoglobin level (13.8 vs. 14.4 g/dl, *p* = 0.001), a higher conversion rate to an open procedure (22 vs. 6%, *p* < 0.001), and more frequent findings of necrosis or gangrene (25 vs. 12%, *p* < 0.001) and perityphlitic abscess (53 vs. 31%, *p* < 0.001) (Table 1).

**Table 1:** Baseline patient characteristics

Patient Characteristics	AB ≤ 3	AB ≥ 4	*p*-value
**Number,** ***n*** **(%)**	226	170	
**Age (years), median (IQR)**	48 (27)	53 (30)	**0.049**
**Gender,** ***n*** **(%)**			0.066
Female	91 (40)	85 (50)	
Male	135 (60)	85 (50)	
BMI* (kg/m ^**2**^ **), median (IQR)**	26.1 (5.8)	25.4 (6.3)	0.746
ASA*, ***n*** **(%)**			**0.012**
I	95 (47)	53 (33)	
II	81 (40)	74 (45)	
III	23 (11)	34 (21)	
IV	3 (2)	2 (1)	
**Active smoker,** ***n*** **(%)**	49 (22)	31 (18)	0.449
**Diabetes,** ***n*** **(%)**	19 (8)	14 (8)	1.000
**Preop. blood results, median (IQR)**			
WBC count (x10^9^/l)	13.5 (5.4)	14.0 (6.2)	0.149
CRP (mg/l)	90 (111)	160 (152)	**< 0.001**
Hemoglobin (g/dl)	14.4 (2.1)	13.8 (2.2)	**0.001**
Creatinine (mg/dl)	0.9 (0.3)	0.8 (0.3)	0.740
**Preop. radiological diagnostic**			
App. diameter (mm), median (IQR)	11 (5)	11 (5)	0.954
Intraabdominal fluid, n (%)	119 (53)	94 (55)	0.612
Appendicolith, n (%)	26 (12)	27 (16)	0.234
**Time to appendectomy**			0.064
≤ 6 h	104 (46)	62 (37)	
> 6 h	122 (54)	108 (63)	
**Time of appendectomy**			0.170
Daytime	89 (39)	55 (32)	
Nighttime	137 (61)	115 (68)	
**Surgical experience**			0.422
Resident	36 (16)	33 (19)	
Specialist	190 (84)	137 (81)	
**Surgical approach,** ***n*** **(%)**			**< 0.001**
Laparoscopic	139 (62)	88 (52)	
Open	73 (32)	44 (26)	
Conversion	14 (6)	38 (22)	
**Duration of surgery (min), median (IQR)**	69 (39)	87 (41)	**< 0.001**
**Need of coecum resection,** ***n*** **(%)**	23 (10)	28 (17)	0.070
**Intraoperative findings,** ***n*** **(%)**			
Perforation	197 (87)	143 (84)	0.467
Necrosis or gangrene	27 (12)	43 (25)	**< 0.001**
Perithyphilitic abscess	71 (31)	90 (53)	**< 0.001**
**AAST classification,** ***n*** **(%)**			**< 0.001**
III	141 (62)	63 (37)	
IV	71 (31)	84 (49)	
V	14 (6)	23 (14)	
**Malignancy,** ***n*** **(%)**	7 (3)	5 (3)	1.000

BMI = body mass index; ASA-score (ASA) = American Society of Anesthesiologists score; WBC = white blood cell, CRP = C-reactive protein

*incomplete data: BMI: *n* = 293; ASA: *n* = 365

### Postoperative outcomes

Intraabdominal abscess and wound infections occurred in 2% and 4%, respectively. The incidence of an intraabdominal abscess (2% vs. 3%, *p* = 0.507) and of wound infections (3% vs. 6%, *p* = 0.080) did not differ significantly between the groups. Patients with a longer duration of postoperative antibiotic therapy showed a higher rate of morbidity (9% vs. 26%, *p* < 0.001) and of need of re-surgery (1% vs. 6%, *p* = 0.011) as well as a longer hospital stay (4 vs. 6 days, *p* < 0.001) (Table 2).

**Table 2:** Postoperative outcome

	AB ≤ 3 (n = 226)	AB ≥ 4 (n = 170)	*p*-value
Morbidity	21 (9)	45 (26)	**< 0.001**
Clavien Dindo			**< 0.001**
I	6 (3)	14 (8)	
II	3 (1)	9 (5)	
III	7 (3)	18 (11)	
IV	3 (1)	4 (2)	
V (Mortality)	2 (1)	0 (0)	
Wound infection	6 (3)	11 (6)	0.080
Intraabdominal abscess	4 (2)	5 (3)	0.507
Paralysis/ileus	1 (0)	4 (2)	0.169
Re-surgery	3 (1)	11 (6)	**0.011**
Length of postoperative hospital stay	4 (2)	6 (4)	**< 0.001**
Readmission (within 90 days)	8 (4)	10 (6)	0.332

### Risk factors for the occurrence of an intraabdominal abscess

Uni- and multivariate analysis revealed the need of a coecum resection as the only independent risk factor for postoperative intraabdominal abscess (OR 5.5 CI (1.4–21.5), *p* = 0.014). There was no association between the duration of postoperative antibiotics and the incidence of a postoperative intraabdominal abscess (Table 3).

**Table 3:** Uni- and multivariate analysis of risk factors for intraadominal abscess

	Univariate	Multivariate		
	*p*-value	OR	CI	*p*-value
Age (high vs. low)*	0.327	-	-	-
Gender (female vs. male)	0.737	-	-	-
BMI (high vs. low)*	1.000	-	-	-
ASA (I/II vs. III/IV)	1.000	-	-	-
Active smoking (yes vs. no)	0.394	-	-	-
Diabetes (yes vs. no)	0.168	-	-	-
Preoperative WBC count (high vs. low)*	0.171	-	-	-
Preoperative CRP (high vs. low)*	0.175	-	-	-
Preoperative hemoglobin (high vs. low)*	0.724	-	-	-
Preoperative creatinine (high vs. low)*	0.725	-	-	-
App. diameter (high vs. low)*	0.578	-	-	-
Intraabdominal fluid in radiological diagnostic (yes vs. no)	0.067	-	-	-
Appendicolith (yes vs. no)	1.000	-	-	-
Time to appendectomy (≤ 6 h vs. > 6 h)	0.740	-	-	-
Time of appendectomy (daytime vs. nighttime)	1.000	-	-	-
Surgical experience (resident vs. specialist)	0.710	-	-	-
Conversion (yes vs. no)	1.000	-	-	-
Coecum resection (yes vs. no)	**0.019**	**5.5**	**1.4–21.5**	**0.014**
Operative time (high vs. low)*	0.335	-	-	-
Intraoperative perforation (yes vs. no)	1.000	-	-	-
Intraoperative necrosis or gangrene (yes vs. no)	0.371	-	-	-
Intraoperative perithyphilitic abscess (yes vs. no)	0.495	-	-	-
AAST classification (III vs. IV vs. V)	0.466	-	-	-
Malignancy (yes vs. no)	1.000	-	-	-
Length of postoperative antibiotics (≤ 3 days vs. ≥ 4 days)	0.507	1.4	0.4–5.5	0.609

* Cut-offs for metric data = median

### Risk factors for the occurrence of a wound infection

For the postoperative incidence of wound infection, five risk factors could be identified in univariate analysis: higher age (*p* = 0.005) and BMI (*p* = 0.035), worse ASA-Score (*p* = 0.014), diabetic patients (*p* = 0.009) and conversion to open procedure (*p* = 0.015). However, only two of these factors were confirmed as independent risk factors in multivariate analysis: higher BMI (OR 5.9 CI (1.2–29.2), *p* = 0.030) and conversion (OR 5.2 CI (1.4–20.0), *p* = 0.016). The duration of postoperative antibiotics showed again no significant impact on the incidence of a postoperative wound infection (Table 4).

**Table 4:** Multivariate analysis of risk factors for wound infection

	Univariate	Multivariate		
	*p*-value	OR	CI	*p*-value
Age (high vs. low)*	**0.005**	2.6	0.6 - 10.7	0.187
Gender (female vs. male)	0.619	-	-	-
BMI (high vs. low)*	**0.035**	**5.9**	**1.2–29.2**	**0.030**
ASA (I/II vs. III/IV)	**0.014**	1.6	0.4–7.1	0.546
Active smoking (yes vs. no)	0.542	-	-	-
Diabetes (yes vs. no)	**0.009**	1.0	0.2–6.0	0.996
Preoperative WBC count (high vs. low)*	1.000	-	-	-
Preoperative CRP (high vs. low)*	0.470	-	-	-
Preoperative hemoglobin (high vs. low)*	0.810	-	-	-
Preoperative creatinine (high vs. low)*	0.076	-	-	-
App. diameter (high vs. low)*	0.393	-	-	-
Intraabdominal fluid in radiological diagnostic (yes vs. no)	0.291	-	-	-
Appendicolith (yes vs. no)	1.000	-	-	-
Time to appendectomy (≤ 6 h vs. > 6 h)	0.803	-	-	-
Time of appendectomy (daytime vs. nighttime)	0.616	-	-	-
Surgical experience (resident vs. specialist)	0.748	-	-	-
Conversion (yes vs. no)	**0.015**	**5.2**	**1.4–20.0**	**0.016**
Coecum resection (yes vs. no)	0.709	-	-	-
Operative time (high vs. low)*	0.468	-	-	-
Intraoperative perforation (yes vs. no)	0.147	-	-	-
Intraoperative necrosis or gangrene (yes vs. no)	0.559	-	-	-
Intraoperative perithyphilitic abscess (yes vs. no)	0.802	-	-	-
AAST classification (III vs. IV vs. V)	0.125	-	-	-
Malignancy (yes vs. no)	1.000	-	-	-
Length of postoperative antibiotics (≤ 3 days vs. ≥ 4 days)	0.080	1.3	0.4–5.0	0.659

* Cut-offs for metric data = median

## Discussion

The use of antibiotics following emergency appendectomy for complicated appendicitis is a common practice, primarily based on intraoperative findings like peritonitis or abscesses. The objective of continued postoperative antibiotic use is to prevent infectious complications, particularly intraabdominal abscesses and wound infections. Nevertheless, there is an ongoing debate regarding the optimal duration of postoperative antibiotic therapy.

Our analysis showed an incidence of an intraabdominal abscess and of wound infections of 2% and 4%, respectively. This corresponds to approximately one-third of all postoperative complications in our cohort and, therefore, demonstrates significant relevance. Other studies have reported similar incidences, ranging from 1.5% up to 7.9% for the occurrence of intraabdominal abscess and 3.4–10.0% for wound infection [17–19, 26].

In our cohort, an extended duration of antibiotic treatment was not associated with a reduced risk of developing an intraabdominal abscess or wound infections. These findings align with recent randomized controlled trials and two other large observational studies: De Wijkerslooth et al. conducted a large randomized controlled trial in 15 hospitals in the Netherlands, randomizing 1066 patients to receive 2 days or 5 days of intravenous postoperative antibiotics after appendectomy. In this trial, 2 days of postoperative intravenous antibiotics for complex appendicitis was non-inferior to 5 days in terms of infectious complications and mortality within 90 days [27]. Liu et al. showed also comparable rates of infectious complications in pediatric patients by comparing 350 patients receiving a fixed 3-days intravenous antibiotic regimen with 336 patients with a prolonged intravenous antibiotic therapy of minimum of 5 days (completed by further oral antibiotic intake for at all 10 days) [28]. In the randomized controlled trial of Saar et al. a 24-hour intravenous antibiotic regimen was compared to an extended antibiotic treatment based on clinical signs. Both patients groups showed with approximately 20% a similar rate of infectious complications. However, the validity of this trial may be limited due to a small sample size (*n* = 80) [29]. van Rossem et al. compared a 3-day course of postoperative anti-biotics to a 5-day course in 267 patients with complicated appendicitis across two Dutch hospitals and found no significant difference between the two groups in terms of intraabdominal abscess or wound infection development. Notably, a laparoscopic approach was identified as a risk factor for intraabdominal abscess in univariate analysis but not in multivariate analysis [17]. The second observational study, conducted by Cho et al., involved 496 patients with complicated appendicitis and similarly demonstrated that pro-longed postoperative antibiotic therapy does not prevent intraabdominal abscess development. This study emphasized the importance of intraoperative source control, which was significantly associated with the occurrence of an intraabdominal abscess [18]. Further studies as well as a recent meta-analysis, which included the two aforementioned large observational studies along with five additional studies (three of which were randomized controlled trials), also concluded that extended postoperative antibiotic use may not be linked to a reduced risk of intraabdominal infection [20, 21, 29, 30]. However, the mentioned meta-analysis acknowledged the limitation of results due to heterogeneity between studies and underpowered trials [20]. Thus, our retrospective large cohort analysis may provide support for previous evidence, even though it is not a randomized controlled trial.

Especifically, the duration of postoperative antibiotic therapy is a topic that complicates the comparability of existing studies, as it varies significantly between studies. We chose a three-day cutoff for antibiotic therapy in our analysis as it appears to be a rational limit for antibiotic treatment and is one of the most used cut-offs in the literature. However, this cutoff is not set in stone and cannot be considered definitive. As a subgroup analysis, we also conducted our results using cutoffs of 0 versus 2–3 versus > 3 postoperative days of antibiotics and obtained the same results as well as the same risk factors. Even the group with no postoperative antibiotic therapy showed no increased risk for intraabdominal abscesses and wound infections. However, the group of patients without postoperative antibiotics comprised only 66 patients, so the currently chosen cutoff pro-vides more statistical power, thereby enhancing the validity of our findings regarding the preference for short-term antibiotic therapy.

The importance of the optimal duration of postoperative antibiotic therapy is underscored by the fact that prolonged postoperative antibiotic therapy carries significant socioeconomic consequences. Several studies have shown that a longer postoperative antibiotic therapy is associated with an extended hospital stay, leading to a significant increase in healthcare costs [21, 22]. Current studies suggest that oral antibiotic therapy may be equivalent to intravenous therapy for postoperative treatment of complicated appendicitis [31]. Nevertheless, patients are typically kept hospitalized for continuation of intravenous antibiotic therapy.

Our risk factor analysis for intraabdominal abscess identified the need for a coecum resection as an independent risk factor, which is a new finding compared to previous studies that did not investigate this parameter. However, the need for a coecum resection typically indicates advanced inflammation, which can reasonably explain the higher risk of developing an intraabdominal infection. In the study by van Rossem, the laparoscopic approach was identified as a risk factor, which was not confirmed in our analysis, consistent with other studies [17, 26, 32, 33]. We also did not investigate incomplete source control as a potential risk factor, as described by Cho et al. [18].

The incidence of wound infection after appendectomy was independently associated with a higher BMI and the need for conversion in our cohort. Both of these parameters are known risk factors for superficial surgical site infections following various abdominal surgeries, including appendectomy [21]. Further risk factors mentioned in the literature but not supported by our analysis include the open approach and the presence of gangrenous appendicitis [34, 35].

There are several limitations to consider in the evaluation of our data. First, the retrospective, single-center design of this study may introduce some bias, although it ensures a consistent surgical and antibiotic therapy for the studied cohort. Second, the primary limitation arises from the selection bias. The individually determined duration of antibiotic therapy led to a situation in which patients with more advanced appendicitis, although all cases were classified as complicated appendicitis, were included in the groups with extended antibiotic regimens. This was because the surgeon’s decision about the duration of postoperative antibiotic therapy is significantly influenced by the pre- and intraoperative findings. This is evident from the differences among the groups, such as higher pre-operative CRP levels, a greater need for conversion and a higher rate of intraoperative gangrene and perityphlitic abscess. However, these variables were included in the risk factor analysis and showed no significant association with intraabdominal abscesses and wound infections. Moreover, patients who suffer from complications during the first three postoperative days and received therefore an extended antibiotic therapy were automatically grouped as extended antibiotic users. This is reflected in the higher rate of postoperative complications and re-surgeries in this group, which could introduce bias into our results. Third, the number of wound infections may be underestimated since such complications are often treated in the outpatient clinic.

## Conclusion

Our data suggest that an extended postoperative antibiotic therapy may not be associated with a reduced risk for the occurrence of an intraabdominal abscess or wound infections. Therefore, short-term antibiotic treatment after appendectomy for complicated appendicitis should be preferred. Patients with the need of a coecum resection or conversion to an open procedure as well as a BMI above 25 kg/m^2^ are more likely to suffer from infectious complications and should receive special attention during postoperative course.

## Data Availability

No datasets were generated or analysed during the current study.

## References

[1] Stewart B, Khanduri P, McCord C, Ohene-Yeboah M, Uranues S, Vega Rivera F, Mock C (2014). Global disease burden of conditions requiring emergency surgery. Br J Surg.

[2] Drake FT, Flum DR (2013). Short- and long-term mortality after appendectomy in Sweden 1987–2006: influence of appendectomy diagnosis, sex, age, co-morbidity, surgical method, hospital volume, and time period–a national population based cohort study. World J Surg.

[3] Bhangu A, Søreide K, Di Saverio S, Assarsson JH, Drake FT (2015) Acute appendicitis: modern understanding of pathogenesis, di-agnosis, and management. Lancet 386(10000):1278–1287. 10.1016/S0140-6736(15)00275-5. Erratum in: Lancet. 2017;390(10104):1736. PMID: 26460662 10.1016/S0140-6736(15)00275-5 26460662

[4] Walędziak M, Lasek A, Wysocki M, Su M, Bobowicz M, Myśliwiec P, Astapczyk K, Burdzel M, Chruściel K, Cygan R, Czubek W, Dowgiałło-Wnukiewicz N, Droś J, Franczak P, Hołówko W, Kacprzyk A, Karcz WK, Kenig J, Konrad P, Kopiejć A, Kot A, Krakowska K, Kukla M, Leszko A, Łozowski L, Major P, Makarewicz W, Malinowska-Torbicz P, Matyja M, Michalik M, Niekurzak A, Nowiński D, Ostaszewski R, Pabis M, Polańska-Płachta M, Rubinkiewicz M, Stefura T, Stępień A, Szabat P, Śmiechowski R, Tomaszewski S, von Ehrlich-Treuenstätt V, Wasilczuk M, Wierdak M, Wojdyła A, Wroński JW, Zwolakiewicz L, Pędziwiatr M (2019) Risk factors for serious morbidity, prolonged length of stay and hospital readmission after laparoscopic ap-pendectomy - results from Pol-LA (Polish Laparoscopic Appendectomy) multicenter large cohort study. Sci Rep 9(1):14793. 10.1038/s41598-019-51172-2. Erratum in: Sci Rep. 2019;9(1):18479. PMID: 31616053; PMCID: PMC6794313 10.1038/s41598-019-51172-2 PMC6794313 31616053

[5] Ward NT, Ramamoorthy SL, Chang DC, Parsons JK (2014) Laparoscopic appendectomy is safer than open appendectomy in an elderly population. JSLS 18(3):e2014.00322. 10.4293/JSLS.2014.00322. PMID: 25392668; PMCID: PMC4208904 10.4293/JSLS.2014.00322 PMC4208904 25392668

[6] Moreira LF, Garbin HI, Da-Natividade GR, Silveira BV, Xavier TV (2018) Predicting factors of postoperative complications in ap-pendectomies. Rev Col Bras Cir 45(5):e19. Portuguese, English. 10.1590/0100-6991e-20181920. PMID: 30462825 10.1590/0100-6991e-20181920 30462825

[7] Zhang P, Zhang Q, Zhao H, Li Y (2020). Factors affecting the length of hospital stay after laparoscopic appendectomy: a single cen-ter study. PLoS ONE.

[8] Patel SV, Nanji S, Brogly SB, Lajkosz K, Groome PA, Merchant S (2018). High complication rate among patients undergoing appen-dectomy in Ontario: a population-based retrospective cohort study. Can J Surg.

[9] Andert A, Alizai HP, Klink CD, Neitzke N, Fitzner C, Heidenhain C, Kroh A, Neumann UP, Binnebösel M (2017). Risk factors for morbidity after appendectomy. Langenbecks Arch Surg.

[10] Bancke Laverde BL, Maak M, Langheinrich M, Kersting S, Denz A, Krautz C, Weber GF, Grützmann R, Brunner M (2023). Risk fac-tors for postoperative morbidity, prolonged length of stay and hospital readmission after appendectomy for acute appendici-tis. Eur J Trauma Emerg Surg.

[11] Varadhan KK, Neal KR, Lobo DN (2012). Safety and efficacy of antibiotics compared with appendicectomy for treatment of uncom-plicated acute appendicitis: meta-analysis of randomised controlled trials. BMJ.

[12] Hansson J, Körner U, Khorram-Manesh A, Solberg A, Lundholm K (2009) Randomized clinical trial of antibiotic therapy versus appendicectomy as primary treatment of acute appendicitis in unselected patients. Br J Surg 96(5):473 – 81. 10.1002/bjs.6482. Erratum in: Br J Surg. 2009;96(7):830. PMID: 19358184 10.1002/bjs.6482 19358184

[13] McCutcheon BA, Chang DC, Marcus LP, Inui T, Noorbakhsh A, Schallhorn C, Parina R, Salazar FR, Talamini MA (2014). Long-term outcomes of patients with nonsurgically managed uncomplicated appendicitis. J Am Coll Surg.

[14] Vons C, Barry C, Maitre S, Pautrat K, Leconte M, Costaglioli B, Karoui M, Alves A, Dousset B, Valleur P, Falissard B, Franco D (2011) Amoxicillin plus clavulanic acid versus appendicectomy for treatment of acute uncomplicated appendicitis: an open-label, non-inferiority, randomised controlled trial. Lancet 377(9777):1573–9. 10.1016/S0140-6736(11)60410-8. PMID: 21550483 10.1016/S0140-6736(11)60410-8 21550483

[15] Salminen P, Paajanen H, Rautio T, Nordström P, Aarnio M, Rantanen T, Tuominen R, Hurme S, Virtanen J, Mecklin JP, Sand J, Jartti A, Rinta-Kiikka I, Grönroos JM (2015) Antibiotic Therapy vs Appendectomy for Treatment of Uncomplicated Acute Appendi-citis: The APPAC Randomized Clinical Trial. JAMA 313(23):2340–8. 10.1001/jama.2015.6154. PMID: 26080338 10.1001/jama.2015.6154 26080338

[16] Wilms IM, de Hoog DE, de Visser DC, Janzing HM (2011) Appendectomy versus antibiotic treatment for acute appendicitis. Cochrane Database Syst Rev (11):CD008359. 10.1002/14651858.CD008359.pub2. Update in: Cochrane Data-base Syst Rev. 2020;10:CD008359. PMID: 22071846 10.1002/14651858.CD008359.pub3 PMC10631378 33001448

[17] van Rossem CC, Schreinemacher MH, van Geloven AA, Bemelman WA, Snapshot Appendicitis Collaborative Study Group (2016). Antibiotic Duration After Laparoscopic Appendectomy for Acute Complicated Appendicitis. JAMA Surg 151(4):323–9. 10.1001/jamasurg.2015.4236. PMID: 26580850 10.1001/jamasurg.2015.4236 26580850

[18] Cho J, Park I, Lee D, Sung K, Baek J, Lee J (2016). Antimicrobial treatment after laparoscopic appendectomy for preventing a post-operative intraabdominal abscess: a prospective cohort study of 1817 patients. Int J Surg.

[19] Jaschinski T, Mosch CG, Eikermann M, Neugebauer EA, Sauerland S (2018). Laparoscopic versus open surgery for suspected appen-dicitis. Cochrane Database Syst Rev.

[20] Ramson DM, Gao H, Penny-Dimri JC, Liu Z, Khong JN, Caruana CB, Campbell R, Jackson S, Perry LA (2021). Duration of post-operative antibiotic treatment in acute complicated appendicitis: systematic review and meta-analysis. ANZ J Surg.

[21] van den Boom AL, de Wijkerslooth EML, Giesen LJX, van Rossem CC, Toorenvliet BR, Wijnhoven BPL (2022). Postoperative antibiotics and Time to Reach Discharge Criteria after Appendectomy for Complex Appendicitis. Dig Surg.

[22] de Wijkerslooth EML, Boerma EG, van Rossem CC, Koopmanschap MA, Baeten CIM, Beverdam FH, Bosmans JWAM, Con-sten ECJ, Dekker JWT, Emous M, van Geloven AAW, Gijsen AF, Heijnen LA, Jairam AP, van der Ploeg APT, Steenvoorde P, Toorenvliet BR, Vermaas M, Wiering B, Wijnhoven BPL, van den Boom AL (2023) APPIC Study Group. 2 days versus 5 days of Postoperative Antibiotics for Complex Appendicitis: cost analysis of a Randomized, non-inferiority trial. Ann Surg 12. 10.1097/SLA.0000000000006089Epub ahead of print. PMID: 37698025

[23] Mouch CA, Cain-Nielsen AH, Hoppe BL, Giudici MP, Montgomery JR, Scott JW, Machado-Aranda DA, Hemmila MR (2020) Validation of the American Association for the Surgery of Trauma grading system for acute appendicitis severity. J Trauma Acute Care Surg 88(6):839–846. 10.1097/TA.0000000000002674. PMID: 32459449 10.1097/TA.0000000000002674 32459449

[24] Dindo D, Demartines N, Clavien PA (2004). Classification of surgical complications: a new proposal with evaluation in a cohort of 6336 patients and results of a survey. Ann Surg.

[25] Horan TC, Gaynes RP, Martone WJ, Jarvis WR, Emori TG (1992). CDC definitions of nosocomial surgical site infections, 1992: a modification of CDC definitions of surgical wound infections. Am J Infect Control.

[26] Athanasiou C, Lockwood S, Markides GA (2017) Systematic Review and Meta-Analysis of Laparoscopic Versus Open Appendicec-tomy in Adults with Complicated Appendicitis: an Update of the Literature. World J Surg 41(12):3083–3099. 10.1007/s00268-017-4123-3. PMID: 28717908 10.1007/s00268-017-4123-3 28717908

[27] de Wijkerslooth EML, Boerma EG, van Rossem CC, van Rosmalen J, Baeten CIM, Beverdam FH, Bosmans JWAM, Consten ECJ, Dekker JWT, Emous M, van Geloven AAW, Gijsen AF, Heijnen LA, Jairam AP, Melles DC, van der Ploeg APT, Steen-voorde P, Toorenvliet BR, Vermaas M, Wiering B, Wijnhoven BPL, van den Boom AL, APPIC Study Group (2023). 2 days versus 5 days of postoperative antibiotics for complex appendicitis: a pragmatic, open-label, multicentre, non-inferiority randomised trial. Lancet.

[28] Liu Q, Hao F, Chen B, Li L, Liu Q, Guo C (2020) Multi-Center Prospective Study of Restrictive Post-Operative Antibiotic Treatment of Children with Complicated Appendicitis. Surg Infect (Larchmt) 21(9):778–783. 10.1089/sur.2019.293. Epub 2020 Mar 4. PMID: 32150521 10.1089/sur.2019.293 32150521

[29] Saar S, Mihnovitš V, Lustenberger T, Rauk M, Noor EH, Lipping E, Isand KG, Lepp J, Lomp A, Lepner U, Talving P (2019) Twen-ty-four hour versus extended antibiotic administration after surgery in complicated appendicitis: A randomized controlled tri-al. J Trauma Acute Care Surg 86(1):36–42. 10.1097/TA.0000000000002086. PMID: 30308538 10.1097/TA.0000000000002086 30308538

[30] Kroon HM, Kenyon-Smith T, Nair G, Virgin J, Thomas B, Juszczyk K, Hollington P (2023). Safety and efficacy of short-course intra-venous antibiotics after complicated appendicitis in selected patients. Acta Chir Belg.

[31] Lipping E, Saar S, Reinsoo A, Bahhir A, Kirsimägi Ü, Lepner U, Talving P (2024). Short postoperative intravenous versus oral anti-bacterial therapy in complicated Acute appendicitis: a Pilot Noninferiority Randomized Trial. Ann Surg.

[32] Li Z, Li Z, Zhao L, Cheng Y, Cheng N, Deng Y (2021). Abdominal drainage to prevent intra-peritoneal abscess after appendectomy for complicated appendicitis. Cochrane Database Syst Rev.

[33] Zamaray B, de Boer MFJ, Popal Z, Rijbroek A, Bloemers FW, Oosterling SJ (2023). AbcApp: incidence of intra-abdominal ABsCesses following laparoscopic vs. open APPendectomy in complicated appendicitis. Surg Endosc.

[34] Danwang C, Bigna JJ, Tochie JN, Mbonda A, Mbanga CM, Nzalie RNT, Guifo ML, Essomba A (2020). Global incidence of surgical site infection after appendectomy: a systematic review and meta-analysis. BMJ Open.

[35] de Wijkerslooth EML, de Jonge J, van den Boom AL, van Geloven AAW, Bemelman WA, Wijnhoven BPL, van Rossem CC, Snapshot Appendicitis Study Group (2019). Postoperative Outcomes of Patients With Nonperforated Gangrenous Appendicitis: A National Multicenter Prospective Cohort Analysis. Dis Colon Rectum 62(11):1363–1370. 10.1097/DCR.0000000000001466. PMID: 31596762 10.1097/DCR.0000000000001466 31596762

